# Biomimetic Filler Strategy for Two-Step Universal Dental Adhesives Using PA–ACP/MSN: Effects on Wettability, Immediate Microtensile Bond Strength, and Cytocompatibility

**DOI:** 10.3390/polym17182501

**Published:** 2025-09-16

**Authors:** Yasir Alnakib, Manhal A. Majeed

**Affiliations:** Department of Restorative and Esthetic Dentistry, College of Dentistry, University of Baghdad, Baghdad 10071, Iraq

**Keywords:** biomimetic dental adhesive, biomimetic adhesive design, resin–dentin bonding, universal dental adhesives, mesoporous silica nanoparticles, polyacrylic acid-stabilized amorphous calcium phosphate

## Abstract

This study evaluated a biomimetic filler strategy for two-step universal dental adhesives by integrating amine-functionalized mesoporous silica nanoparticles (MSNs) loaded with polyacrylic acid-stabilized amorphous calcium phosphate (PA–ACP) into the primer phase. MSNs were synthesized and characterized by FTIR, N_2_ sorption (BET), and HRTEM to confirm structural integrity and effective PA–ACP loading. Two commercial adhesives (G2 Bond and OptiBond eXTRa) were modified by incorporating different volumes fractions (10, 15, 20 vol%) of PA–ACP/MSN. Wettability (contact angle), microtensile bond strength (μTBS), and cytotoxicity (indirect MTT assay using human periodontal ligament fibroblasts, HPLFs) were assessed. The results demonstrated that incorporating up to 15 vol% PA–ACP/MSN maintained favorable wettability and bond strength, comparable to those of the unmodified controls. At 20 vol%, significant increases in contact angles and reductions in bond strength indicated impaired primer infiltration. Cytotoxicity testing confirmed high fibroblast viability (>70%) across all tested concentrations, verifying the biocompatibility of PA–ACP/MSN-filled primers. This work confirms the feasibility of a biomimetic adhesive design using PA–ACP/MSN in the primer phase without compromising immediate wettability and immediate μTBS up to 15 vol%. Remineralization is a potential capability that requires verification in future studies.

## 1. Introduction

Resin–dentin bonding is inherently prone to degradation over time, which jeopardizes the longevity of composite restorations. The hybrid layer created by the adhesive infiltration of demineralized dentin is often regarded as the weakest link of the bonded interface, and its gradual breakdown can lead to loss of bond strength and failure of the restoration [1,2,3]. Water sorption and enzymatic activity in the oral environment cause the hydrolysis of hydrophilic resin components and activate matrix metalloproteinases (MMPs) that can degrade exposed collagen fibrils within the hybrid layer [1,4]. Consequently, resin–dentin bond strength deteriorates with aging, leading to the formation of interfacial gaps. These phenomena contribute to microleakage and secondary caries, which remain major causes of restoration failure and replacement [2,5]. Improving the durability of adhesive interfaces is therefore a critical goal; one promising approach is the development of bioactive adhesive systems that can actively participate in remineralizing and protecting the tooth–restoration interface.

Bioactive dental adhesives are formulated to release therapeutic agents that combat degradation and promote tissue repair. Unlike conventional inert adhesives, bioactive adhesives can remineralize adjacent tooth structure, neutralize acids, or even exhibit antibacterial effects [6,7]. For example, incorporating calcium phosphate fillers, fluoride-releasing components, or antibacterial monomers into adhesives has been shown to endow them with the ability to regenerate mineral and inhibit bacterial growth at the bonded interface [6,7,8,9]. Prior studies have reported resin composites and adhesives containing amorphous calcium phosphate (ACP) or bioactive glass fillers that continuously release calcium (Ca^2+^) and phosphate (PO_4_^3−^) ions, leading to the precipitation of new apatite mineral within surrounding demineralized dentin [10,11]. These bioactive modifications aim to mitigate the inherent problem of adhesive degradation by reinforcing the resin–dentin interface over time. By supplying ions that can recrystallize into hydroxyapatite, such adhesives may counteract collagen matrix breakdown and fill nanoscopic voids, thus preserving bond integrity [10]. This strategy is inspired by biomimetic remineralization, where materials are designed to mimic the natural mineralization processes of dental tissues.

In natural enamel and dentin formation, noncollagenous proteins guide the deposition of minerals via amorphous precursor phases. Specifically, enamel biomineralization follows a nonclassical crystallization pathway: calcium and phosphate ions first aggregate into amorphous calcium phosphate clusters, which are stabilized by matrix molecules (such as amelogenin) before they crystallize into ordered apatite structures [12]. Emulating this process, researchers have developed amorphous calcium phosphate-based remineralization systems for dental applications. ACP is a highly soluble precursor that can release Ca^2+^ and PO_4_^3−^ ions to form apatite; however, in isolation, ACP is unstable and tends to rapidly crystallize or aggregate, losing its efficacy [13]. To overcome this, polyelectrolyte stabilizers like polyacrylic acid (PA) are used to stabilize ACP in a metastable state. PA is rich in carboxylate groups that strongly bind Ca^2+^, forming COO^−^–Ca^2+^ complexes that inhibit the premature crystallization of ACP [13,14]. This polyacrylic acid-stabilized amorphous calcium phosphate (PA–ACP) remains amorphous and nanoscale, providing a ready reservoir of remineralizing ions. When exposed to an aqueous environment (such as the poorly resin-impregnated collagen matrix), PA–ACP can release Ca^2+^ and PO_4_^3−^ ions that diffuse into demineralized collagen matrices and precipitate as new hydroxyapatite, thereby remineralizing the tissue in a biomimetic manner [14,15,16]. Importantly, polyacrylic acid is analogous to the polycarboxylate components of glass ionomer cements and has a long history of safe dental use, suggesting that PA–ACP complexes are likely to be biocompatible. Recent studies have shown that incorporating PA–ACP nanoparticles into dental adhesives can impart remineralization capabilities without significant cytotoxicity. For instance, Wang et al. first showed that adding PA–ACP to an experimental adhesive drove intrafibrillar apatite formation and partially restored the mechanical properties of etched dentin, confirming the concept of biomimetic remineralizing adhesives [11]. Nevertheless, the direct dispersion of PA–ACP in resin still suffers from agglomeration and uncontrolled release.

Another emerging strategy is to use mesoporous nanocarriers to deliver remineralizing agents in adhesives. Mesoporous silica nanoparticles (MSNs) demonstrate a high surface area and a network of nanoscale pores that can be loaded with various therapeutic substances [17]. In dentistry, MSNs have been explored as delivery vehicles for calcium, phosphate, fluoride, and antimicrobial agents, owing to their tunable porosity, biocompatibility, and tailorable surface chemistry [18]. These nanocarriers can be engineered to provide the controlled, sustained release of ions or drugs at the site of interest [19]. Notably, loading ACP into mesoporous silica has been shown to improve the stability and efficacy of the remineralizing agent. Hua et al. loaded PA-stabilized ACP into etched mesoporous silica; the hybrid nanoparticle slurry (PA–ACP@MSN) effectively promoted enamel subsurface lesion remineralization in vitro [14]. The mesoporous silica framework likely protects the ACP from premature dissolution or aggregation, while its open porosity allows for sustained ion release when triggered by an acidic environment. MSNs are also advantageous for incorporation into resins due to their silica composition (which can strengthen the material like conventional silica fillers) and their proven cytocompatibility in oral applications [18]. Previous reports have incorporated mesoporous bioactive glass or silica fillers into dental polymers to impart ion release and observed enhanced remineralization of adjacent dentin and resistance to secondary caries without harming the material’s mechanical properties [20,21,22]. Given these benefits, combining PA–ACP with an MSN carrier presents a promising design for a new remineralizing adhesive filler. To date, no dental adhesive has incorporated a nanocomposite that combines polyacrylic acid-stabilized amorphous calcium phosphate (PA–ACP)-loaded mesoporous silica nanoparticles (MSNs).

The present work modified the primer of two different two-step universal adhesive systems with PA–ACP/MSN and assessed their performance in terms of dentin surface wettability (contact angle) and microtensile bond strength (μTBS) relative to unfilled controls, together with testing their biocompatibility through an indirect cytotoxicity test against human periodontal ligament fibroblasts (HPLFs). The objective of this study was to determine whether integrating PA–ACP/MSN into a dental adhesive could impart biomimetic remineralization capabilities without compromising key functional properties, thereby establishing a platform for next-generation adhesives that actively reinforce and protect the bonded interface over time.

## 2. Materials and Methods

### 2.1. Materials

Triethanolamine (TEA), cetyltrimethylammonium bromide (CTAB), tetraethyl orthosilicate (TEOS), 3-aminopropyltriethoxysilane (APTES, MW 221.37 mol^−1^), polyacrylic acid (MW 1800 mol^−1^), calcium chloride dihydrate (CaCl_2_·2H_2_O), dipotassium phosphate (K_s_HPO_4_), and sodium dihydrogen phosphate (NaH_2_PO_4_) were purchased locally from a distributor (Sigma Aldrich, Merck Group, Darmstadt, Germany).

Ethanol and acetone (Lida chemicals, Shijiazhuang, Hebei Province, China) were obtained locally at the university.

Dental materials: Phosphoric acid gel (Kerr Dental, Brea, CA, USA); G2 Bond Universal (GC America, Alsip, IL, USA); OptiBond eXTRa Universal (Kerr Dental, Brea, CA, USA); and dental composite (G–ænial Posterior, GC America, Alsip, IL, USA)—all purchased from their respective local distributors.

### 2.2. MSN Synthesis

MSNs were prepared using the customized templated triethanolamine sol–gel catalysis method reported by Moeller and co-workers [23,24,25]. Synthesis started by dispersing CTAB (1.5 g) in deionized water (74.4 mL) and ethanol (10.5 mL) under magnetic stirring (≈700 rpm, 10 min) to establish micellar templates. Triethanolamine (TEA, 5.2 mL) was then introduced, and the mixture was heated up to 60 °C. Tetraethyl orthosilicate (TEOS, 6.6 mL) was added dropwise and the reaction was maintained for 2 h (60 °C, 700 rpm). The molar ratio of reactants (TEOS/TEA/CTAB/ethanol/H_2_O) was 1:1.33:0.14:6.1:140. The suspension was centrifuged (15,000 rpm, 15 min), and the pellets were triple-washed using 1:1 ethanol/water and air-dried overnight. Finally, calcination was performed to remove the organic template by ramping at 5 °C min^−1^ and then holding at 500 ± 20 °C for 2 h, yielding template-free MSNs for subsequent drug loading [25,26].

### 2.3. Amine Functionalization of the MSNs

Controlled release from mesoporous carriers depends on the electrostatic attraction between the payload and particle surface [27,28]. Although PA–ACP can be loaded into MSNs via capillary imbibition, both species possess negative zeta potentials, favoring rapid-burst release [26,29,30]. To introduce positive charges, template-free MSNs (1 g) were ultrasonicated (MTI Corporation, Richmond, CA, USA) in ethanol (200 mL, 10 min), followed by the dropwise addition of 2 mL of APTES. The suspension was stirred at room temperature for 24 h and then centrifuged (15,000 rpm), washed, and dried overnight, yielding amine-grafted MSNs (AF–MSNs) suitable for electrostatically moderated drug delivery [26,31].

### 2.4. PA–ACP Synthesis and Loading into AF-MSNs

Equal volumes of 13.5 mM CaCl_2_·2H_2_O (25 mL) and 6.3 mM K_2_HPO_4_ (25 mL) stock solutions, each containing polyacrylic acid (500 µg mL^−1^), were mixed under magnetic stirring to generate a polymer-induced liquid precursor (PILP) of PA–ACP [32,33,34]. The initial pH (≈8.4) was adjusted with 1 molar NaH_2_PO_4_ dropwise to reach the target pH of 7.4.

AF–MSNs (300 mg) were dispersed in 50 mL of the PA–ACP suspension, sonicated on ice for 12 2.5-min cycles to ensure proper nanoparticle dispersion. The mixture was then gently stirred for 24 h to promote capillary imbibition and then left undisturbed for 48 h to complete loading. Particles were recovered by centrifugation (15,000 rpm, 15 min), air-dried, and resuspended in acetone (2 mL) using a vortex mixer (3000 rpm, 5 min) (DLAB, Beijing, China) to yield a stable PA–ACP/MSN colloidal suspension for subsequent adhesive incorporation.

### 2.5. Physicochemical Characterization of MSNs

Fourier-transform infrared spectroscopy (FTIR), N_2_ sorption (BET), and high-resolution transmission electron microscopy (HRTEM) were used to confirm silica network formation, surfactant removal, amine grafting and drug loading.

FTIR (8400S, Shimadzu, Kyoto, Japan): wavenumber spectra (400–4000 cm^−1^) were collected on KBr pellets (sample/KBr = 1:100, wt/wt). Peaks at ≈1080/810/460 cm^−1^ (Si–O–Si), 2920/2850 cm^−1^ (CTAB C-H) and 1550 cm^−1^ (NH_2_ bending) were monitored to verify silica condensation, template removal, and amine functionalization, respectively [29,35].

Brunauer–Emmett–Teller (BET) (ASAP, Micrometrics, Norcross, GA, USA): AF–MSNs (200 mg) were outgassed at 150 °C (12 h) and analyzed using a BET analyzer (ASAP 2010, Micromeritics, Norcross, GA, USA). Nitrogen adsorption–desorption isotherms (77 K) provided specific surface area (BET) and pore metrics via Barrett–Joyner–Halenda (BJH) analysis, confirming mesoporosity essential for drug loading [27,36,37].

HRTEM (JEM–ARM300F, JEOL, Tokyo, Japan): Unloaded MSNs and PA–ACP-loaded MSNs (acetone colloid) were drop-cast onto Lacey carbon-coated Cu grids (300-mesh), air-dried 60 min, and vacuum-dried before imaging. Micrographs were processed in ImageJ software Version 1.54p (National Institutes of Health (NIH), Bethesda, MD, USA) to determine particle size, pore ordering, and intra-particle PA–ACP deposition [26,30,38].

### 2.6. Incorporation of PA–ACP/MSN into Two-Step Universal Adhesive Primers

The PA–ACP/MSN colloid was blended into the primer phases of two commercial two-step universal adhesive primers: G2 Bond “G2” (GC America, Alsip, IL, USA) and OptiBond eXTRa “OE” (Kerr Dental, USA). Filler content was defined by volume fraction to avoid discrepancies arising from the different densities and viscosities of the two primers, a practice recommended in nanofiller studies of dental resins [39,40,41]. Volume fraction was calculated using the following formula:

Volume fraction = [V_NP_/(V_NP_ + V_Primer_)] × 100% (1)

where V_NP_ is the volume of nanoparticle colloid (µL) and V_Primer_ is the volume of the primer (µL).

Target loadings of 10, 15, and 20 vol% were achieved by pipetting 100, 150, or 200 µL of nanofiller suspension into 900, 850, or 800 µL of primer, respectively. Each mixture was vortexed (3000 rpm, 5 min) to ensure homogeneous dispersion before use.

### 2.7. Preliminary Studies

#### 2.7.1. Wettability (Contact Angles) Study

Static contact angles were determined with an optical tensiometer (Ossila, Creating Nano Technologies Inc., Tainan City, Taiwan) using the sessile-drop protocol [42,43]. Flat mid-coronal dentin slabs were mounted perpendicular to the camera axis and aligned with the instrument background. Then, 5 µL droplets of each control and PA–ACP/MSN-modified primer (10, 15, 20 vol%) were dispensed onto the surfaces (n = 3 dentin slabs per formulation; 3 drops per slab). The instrument’s software fitted the drop profile (Young–Laplace) and recorded the contact angle within 30 s of deposition, providing comparative measures of the primers’ wettability.

#### 2.7.2. Microtensile Bond Strength (μTBS) Study

Testing followed the Academy of Dental Materials protocol and ISO/TS 4640:2023 [44,45]. Twenty-four sound human premolars were sectioned 3 mm below the cusp tip with a low-speed diamond saw (SYJ-150, MGSI Lab Equipment’s, Shanghai, China) to expose flat mid-coronal dentin. The exposed dentin surfaces were polished with ascending silicon carbide papers (Struers, Ballerup, Denmark) up to 600 grit size. Six teeth were bonded with unmodified primers/adhesives (control), and eighteen tooth samples were treated with PA–ACP/MSN-modified G2 and OE primers at 10, 15, or 20 vol% followed by adhesive placement. Composite buildups were carried out with dental composite (G-ænial Posterior, GC America, Alsip, IL, USA) (≤2 mm increments, total 4 mm), and each increment was light-cured (Valo, Ultradent Inc., South Jordan, UT, USA) for 20 s. The samples were stored in simulated body fluid at 37 °C for 24 h, and then sectioned into 1×1 mm adhesive-stick specimens (five per tooth; n = 15 per group).

Each adhesive-stick specimen was glued (cyanoacrylate) from both ends to custom-made tensile grip, kept moist, and loaded in tension in a horizontal universal tester (ZQ-780, Laryee, Beijing, China) at a crosshead speed of 0.7 mm min^−1^ until failure. μTBS values (MPa) were calculated as follows:

μTBS (MPa) = P/A(2)

where P is the load at failure (Newton) and A is the bonded area (mm^2^).

Failure modes were classified under ×20 magnification as adhesive, mixed, or cohesive.

### 2.8. Indirect MTT Cytotoxicity Assay

The 15 vol% PA–ACP/MSN adhesive formulations were screened for biocompatibility on human periodontal ligament fibroblasts (HPLFs) by the indirect extracts method, as described by ISO 7405:2025 and ISO 10993–5:2009 guidelines [46,47,48].

Disc fabrication: Primer and adhesive were mixed at a 1:1 ratio and injected into a circular PTFE mold (Ø 5 mm × 2 mm), air-thinned, and light-cured for 20 s on each side and polished with 1500-grit SiC paper. Eighteen discs per group (G2-C, G2-15%, OE-C, and OE-15%) and six discs per subgroup were allocated to each extraction time (24 h, 48 h, and 72 h). The specimens were UV-sterilized for 30 min per side in a laminar hood [49,50].

Extract preparation: Six discs (total surface area = 4.24 cm^2^) were immersed in 1.5 mL serum-free DMEM (≈2.8 cm^2^ mL^−1^) with intermittent agitation at 37 °C and 5% CO_2_ for 24 h, 48 h, or 72 h. Eluates were filtered (0.22 µm syringe filter, Millex™, Merck, Darmstadt, Germany) and stored at −20 °C; medium alone served as the negative control [49].

Cell culture: HPLFs (ScienCell Research Laboratories, Carlsbad, CA, USA) were expanded in DMEM + 10% FBS, 100 U mL^−1^ penicillin, 100 µg mL^−1^ streptomycin, and 2 mM L-glutamine at 37 °C and 5% CO_2_ to 80% confluence. Cells were detached (0.25% trypsin-EDTA), counted with a hemocytometer, and seeded at 1 × 10^5^ cells mL^−1^ in 96-well plates (200 µL well^−1^) [51].

MTT assay. After 24 h pre-incubation, the culture medium was replaced with 200 µL of each extract (in triplicate). Following a 24 h exposure, 10 µL of MTT solution was added; the plates were incubated for a further 4 h, the medium was removed, and formazan was solubilized (100 µL). Absorbance was read at 575 nm [49,52]; the cell viability % was calculated as follows:

Cell Viability (%) = (OD_sample_/OD_control_) × 100% (3)

where OD is the optical density of the measured samples and controls, respectively.

Materials were classed as noncytotoxic when the viability remained at ≥70%, in line with ISO 10993-5 criteria.

### 2.9. Statistical Analysis

The results are presented as mean ± standard deviation. Analyses were run in IBM SPSS, version 21. Data normality (Kolmogorov–Smirnov, Shapiro–Wilk) and homogeneity (Levene test) were confirmed (α = 0.05). Contact angle and μTBS means: one-way ANOVA was applied, factor: nanofiller level (C, 10, 15, 20 vol%), followed by a Dunnett post hoc test to compare each incorporation percentage directly against the unmodified control. MTT viability was evaluated by two-way ANOVA, factors: adhesive type (G2 vs. OE) and nanofiller loading (control vs. 15 vol%) for each immersion time (24 h, 48 h, 72 h); Bonferroni post hoc tests were used estimated marginal means. A global significance level of *p* < 0.05 was adopted for all tests.

## 3. Results

### 3.1. Physicochemical Characterization

#### 3.1.1. FTIR Spectroscopy

**Figure 1:**
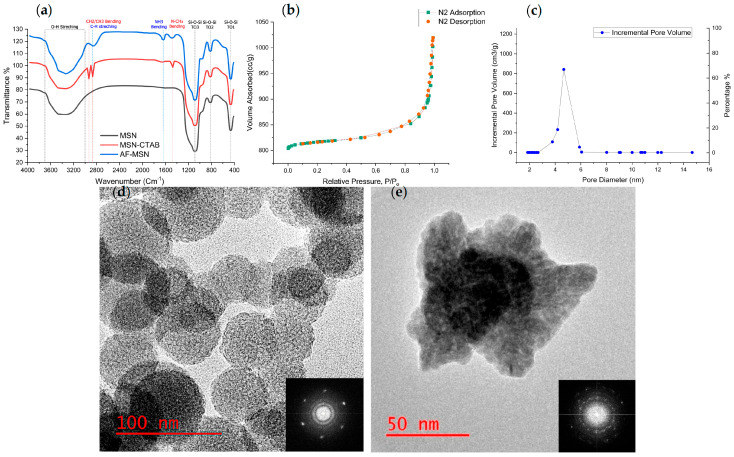
Physicochemical validation of AF–MSNs and subsequent PA–ACP loading. (**a**) FTIR spectra illustrating (i) CTAB-templated MSNs, (ii) template-free MSNs, and (iii) amine-grafted MSNs; disappearance of CH_2_/CH_3_ and N-CH_3_ bands verifies surfactant removal, while the new N–H band confirms amine coupling. (**b**) Type IV N_2_ adsorption–desorption isotherm with H1 hysteresis, characteristic of uniform mesoporosity. (**c**) BJH pore size distribution centered at 4.8 nm, indicating a narrow mesopore window. (**d**) HRTEM image of template-free MSNs (scale = 100 nm) showing spherical particles with radially ordered pores; FFT inset demonstrates mesoscopic ordering within an amorphous matrix. (**e**) HRTEM of PA–ACP-loaded AF–MSNs (scale = 50 nm) revealing a pore-filled yet structurally intact particle; FFT inset retains amorphous signature, indicating ACP remains noncrystalline.

Figure 1a compares the spectra of CTAB-templated MSNs, template-free MSNs, and amine-grafted MSNs (400–4000 cm^−1^, transmission mode). All samples display the characteristic silica framework bands [29]: Si–O–Si asymmetric stretch (≈1080 cm^−1^, TO_3_), symmetric stretch (≈810 cm^−1^, TO_2_) and bending mode (≈460 cm^−1^, TO_1_) and a broad 3000–3700 cm^−1^ envelope assigned to surface silanol and adsorbed water.

Surfactant removal: The methylene/methyl stretches of CTAB at 2920–2950 and 2850 cm^−1^ and the N–CH_3_ bending at 1480 cm^−1^ disappear after calcination, confirming efficient template extraction.

Amine grafting: AF-MSNs show a new N–H scissoring band at 1630 cm^−1^ and weak C–H stretches at ≈2835 cm^−1^, evidencing successful APTES coupling without perturbing the silica network.

#### 3.1.2. BET

AF-MSNs display a type-IV isotherm with H1 hysteresis (Figure 1b), typical of uniform mesoporous silica [53]. The BET surface area is 861 m^2^ g^−1^ (single-point ≈ 858 m^2^ g^−1^; t-plot external ≈ 867 m^2^ g^−1^), within the 600–1000 m^2^ g^−1^ range reported for aminated MSNs [19,54,55]. BJH pore-size distribution (Figure 1c) is narrowly centered at 4.8 nm, and the cumulative pore volume reaches 0.69 cm^3^ g^−1^, confirming a well-developed, uniform mesopore network. A minor microporous contribution (≈55 m^2^ g^−1^, Dubinin–Astakhov) is consistent with previous reports for post-grafted MSNs [56,57].

#### 3.1.3. HRTEM

Representative micrographs (Figure 1d) revealed quasi-spherical particles (53.2 ± 8.7 nm; ImageJ analysis) with radially oriented mesopores and rough peripheries. Fast Fourier transform (FFT) patterns exhibit discrete ring reflections superimposed on a diffuse halo, indicative of ordered pore periodicity embedded in an amorphous silica matrix.

After PA–ACP loading (Figure 1e), individual particles appear less aggregated and a faint shell of electron-dense material lines the pore walls, consistent with capillary imbibition of the liquid precursor. FFT images retain the diffuse amorphous signature, confirming that ACP remains noncrystalline and the mesostructure order of the carrier is preserved.

Taken together, the FTIR, HRTEM, and BET analyses demonstrate that CTAB removal, amine functionalization, and PA–ACP loading were achieved without the loss of pore order or excessive pore blockage, yielding a high-surface-area carrier compatible with PA–ACP loading and potential ion release.

### 3.2. Wettability (Contact Angle)

The static contact-angle preliminary data (Table 1) show that baseline wettability differed markedly between the two primers: OE-C produced a very low θ of 9.6° ± 1.9°, whereas the corresponding G2-C yielded 20.6° ± 2.9°.

For G2, adding 10 vol% or 15 vol% nanofiller did not significantly alter wettability (23.4 ± 2.6° and 27.0° ± 2.0°, respectively; *p* > 0.05, Dunnett test). In contrast, 20 vol% PA–ACP/MSN increased θ to 41.8° ± 3.4°, a significant rise versus the control (*p* < 0.05) that indicates impaired spreading.

Similarly, in the OE groups, nanofiller concentrations up to 15 vol% maintained favorable wetting (θ = 12.7° ± 1.1° and 15.4° ± 3.3°; both *p* > 0.05). Only the 20 vol% formulation significantly reduced wettability, elevating θ to 24.3° ± 2.5° (*p* < 0.05). Despite this increase, the absolute value remained well below 30°, a threshold generally regarded as acceptable for primer infiltration into dentin.

Overall, the data confirm that PA–ACP/MSN incorporation up to 15 vol% does not compromise primer wettability, whereas a 20 vol% load negatively affects spreading for both adhesives—more severely for G2 than for OE.

### 3.3. Microtensile Bond Strength (μTBS)

**Figure 2:**
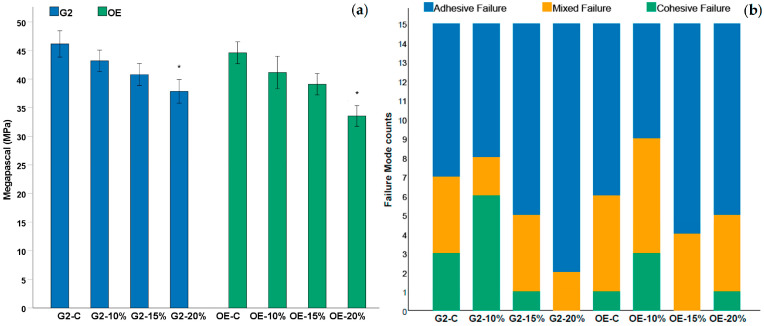
(**a**) Immediate microtensile bond strength (μTBS, mean ± SD; n = 15 sticks per group) of G2 and OE primers containing 0–20 vol% PA–ACP/MSN. Asterisks indicate a significant reduction compared with the respective control (*p* < 0.05, Dunnett post hoc). (**b**) Corresponding failure-mode distributions (adhesive, mixed, cohesive) for each group (15 sticks per group). Both adhesives maintained μTBS up to 15 vol% nanofiller, whereas 20 vol% produced lower bond strength and a marked shift toward adhesive failures.

The preliminary results of μTBS values are summarized in Table 1 and Figure 2a. For the G2 group, the control beams reached 46.2 ± 6.9 MPa. The incorporation of 10 vol% or 15 vol% PA–ACP/MSN produced no significant reductions (43.2 ± 5.7 MPa and 40.8 ± 5.8 MPa, respectively; Dunnett *p* > 0.05). At 20 vol%, the mean bond strength fell to 37.9 ± 6.2 MPa, a significant decrease compared to the control (*p* < 0.05).

A comparable trend was observed for OE groups. The unmodified primer yielded 44.6 ± 5.7 MPa, whereas 10 vol% and 15 vol% nanofiller maintained statistically similar strengths (41.2 ± 8.6 MPa and 39.1 ± 5.6 MPa, *p* > 0.05). Only the 20 vol% formulation exhibited a significant drop to 33.6 ± 4.4 MPa (*p* < 0.05).

A failure mode analysis (Table 1; Figure 2b) (adhesive/mixed/cohesive) revealed a shift toward adhesive failures at the 20 vol% level for both systems (G2-20%: 13/2/0; OE-20%: 10/4/1), consistent with the lower μTBS values. In contrast, the control and ≤15 vol% groups showed a more balanced distribution, with a higher proportion of mixed failures.

Collectively, these results indicate that PA–ACP/MSN loadings up to 15 vol% preserve immediate bond strength, while a 20 vol% filler level compromises interfacial integrity for both universal adhesives. As a pilot adhesive design study, these μTBS values reflect immediate performance; durability will be evaluated in subsequent work.

### 3.4. MTT Assay

**Figure 3:**
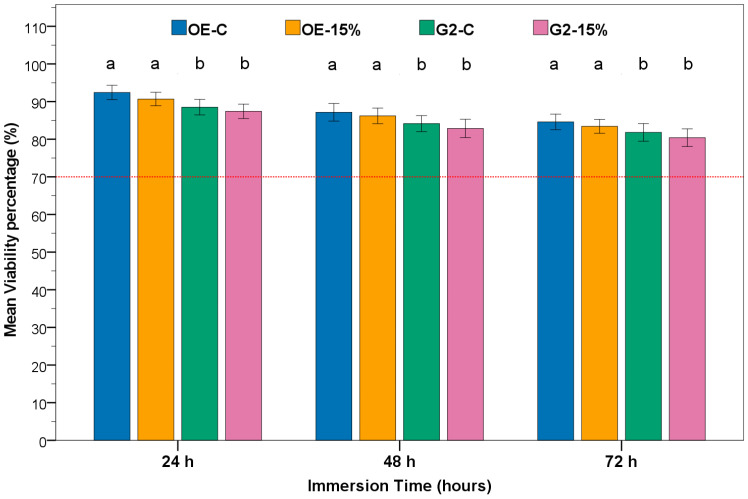
Viability of human periodontal ligament fibroblasts exposed to adhesive extracts (mean ± SD, n = 6 discs → 1 extract, assayed in triplicate wells). Bars show mean cell viability (%) after 24 h, 48 h, and 72 h for adhesive type (G2 and OE) and nanofiller loading (C, 15%). The red dotted line marks the ISO 10993–5:2009 noncytotoxic threshold (70%). Within each time point, bars that do not share the same lowercase letter differ significantly (*p* < 0.05; two-way ANOVA, factors = adhesive type× nanofiller loading, Bonferroni post hoc on estimated marginal means).

Fibroblast viability remained high for every extract, ranging from 92.4 ± 1.8% at 24 h to 80.4 ± 2.1% at 72 h, and never dropped below the ISO 10993-5 noncytotoxic threshold of 70% (Figure 3; Table S1). Two-way ANOVA tables applied separately to each time point identified adhesive type as the only significant factor (Tables S2–S4), with OE consistently supporting greater cell viability than G2. Furthermore, neither the 15 vol% PA–ACP/MSN loading nor the adhesive × nanofiller loading interaction reached significance (*p* > 0.05). Bonferroni-adjusted pairwise tests confirmed that nanofiller incorporation did not alter viability within either adhesive family (OE-C vs. OE-15%, G2-C vs. G2-15%; *p* > 0.05) (Figure 2). These results demonstrate that the PA–ACP/MSN nanofiller is biologically innocuous at the tested concentration and that OE outperforms G2 in maintaining metabolic activity while retaining full cytocompatibility.

## 4. Discussion

This study introduces a novel design for creating biomimetic dental adhesives that integrates amine-functionalized mesoporous silica nanocarriers loaded with polyacrylic-acid-stabilized amorphous calcium phosphate (PA–ACP). Material characterization confirmed that the MSN framework retained its mesostructure integrity and high surface area after functionalization and loading, which is crucial for effective ion delivery [26,58]. FTIR spectra showed the disappearance of the CTAB surfactant’s C–H bands and the appearance of new N–H and C–H signals after APTES treatment. This amine functionalization is important as it provides positively charged sites on the MSNs surface to attract the polyanionic PA–ACP complexes [26,30,59]. Nitrogen sorption analysis revealed a type-IV isotherm with H1 hysteresis—evidence of uniform mesoporosity—with a high surface area, substantial pore volume, and a 4.8 nm pore diameter. These values are consistent with other amino-functionalized MCM-41-type silica nanoparticles in the literature [26,30,38]. HRTEM confirmed the porous architecture and mineral loading. Unloaded AF–MSNs showed wormhole-like mesopore channels, indicating an ordered nanostructure. After PA–ACP loading, the particles exhibited an electron-dense “shell” of mineral with a granular surface, and the internal pore pattern became obscured. This morphology, also reported by Zhang et al., indicates that amorphous calcium phosphate infiltrated the mesopores and coated the nanoparticle surfaces [26]. An FFT analysis of loaded MSNs confirmed that the calcium phosphate remained amorphous (noncrystalline). Maintaining PA–ACP in an amorphous state is crucial for remineralization. In principle, PA–ACP can release Ca^2+^ and PO_4_^3−^ ions that, together with polyacrylic acid, may nucleate new apatite in situ. However, the present study did not demonstrate dentin remineralization; these outcomes remain to be verified in dedicated assays.

Several studies showed that MSNs carrying calcium and phosphate precursors can induce intrafibrillar remineralization in a reconstituted 2D collagen model [26,29,38]. Moreover, the nanoparticles’ small size (≈50 nm) may facilitate infiltration into demineralized dentin and collagen networks, delivering precursors where needed [60]. In the present work, the nanofiller was incorporated in the primer phase to allow the PA–ACP/MSN particles to diffuse into and interact with the exposed collagen network, delivering ions directly to the substrate. Accordingly, our contribution is an integrated design that embeds PA–ACP/MSN in the primer to potentially enable biomimetic remineralization at the hybrid layer without extra clinical steps; this intended function requires confirmation in future remineralization and durability assays.

In our wettability findings, up to 15 vol% PA–ACP/MSN preserved a low static contact angle (<30°) and bond strength. At 20 vol%, the contact angles rose significantly, demonstrating viscosity-driven crowding that may impede resin impregnation and hybrid layer formation. Comparable thresholds have been reported: Pires-de-Souza et al. showed that self-etch adhesive maintained dentin wettability at 2–5 wt% biosilicate nanoparticles but showed higher angles and lower bond strength at 10 wt% [61]. Likewise, Han et al. observed a progressive increase in water contact angle when Zn-N-doped TiO_2_ was blended into a universal adhesive; the bond strength remained clinically acceptable until the highest loading (7 wt%), where a further rise in viscosity coincided with weakened adhesion [62]. Overall, the 15 vol% PA-ACP/MSN incorporation appears a practical ceiling: below this, fillers can add bioactivity without sacrificing flow; above it, viscosity and contact angle rise and hybrid layer integrity may be compromised.

The μTBS data mirrored the wettability findings. 10 vol% and 15 vol% PA–ACP/MSN delivered μTBS values equivalent to controls, indicating that the filler can be incorporated without weakening the resin–dentin bond. Our findings confirm that a moderate loading of MSN-based nanofiller does not hinder resin infiltration or hybrid layer formation, while excessive nanoparticle loading can embrittle the adhesive film and disrupt micromechanical interlocking. This aligns with previous work showing that functionalized MSNs can be added to adhesive primers without compromising bond strength to dentin [22]. Alkhazaleh et al. demonstrated that an experimental primer with Proanthocyanidin-loaded MSNs achieved comparable immediate bond strength relative to unfilled primer [22]. In addition, load dependence is commonly reported in the literature. For example, Yan et al. reported that adding ≥5 wt% Chlorhexidine-encapsulated MSNs into dental adhesive significantly reduced immediate μTBS values [63], whereas Melo et al. preserved bond strength using an unfilled primer phase and a highly filled (≈40 wt%) nano-ACP adhesive [64]. Thus, the acceptable filler threshold depends on where and how the bioactive phase is introduced. When confined to the primer, ≈15 vol% appears optimal; adding fillers to the adhesive phase may allow higher overall loads without compromising bonding performance.

All formulations in this study maintained HPLFs viability well above the 70% threshold for cytotoxicity [51,65], indicating broad in vitro biocompatibility despite the potential for monomer leaching in resin adhesive materials [66,67]. For example, Demirel et al. observed extract-induced viabilities ranging from 60% to 90% for various dental adhesives, depending on their composition [68], whereas in our study, no group fell into the cytotoxic ranges. Notably, the two adhesive formulations differed: the OE consistently yielded higher cell viabilities than the G2 at all time points, although both remained noncytotoxic, possibly reflecting compositional differences. OE contains GPDM as its functional phosphate monomer (along with HEMA and other co-monomers), whereas G2 Bond Universal uses a cocktail of 10-MDP, 4-META, and related monomers (and is HEMA-free). Indeed, such differences are critical, as recent data suggest that GPDM is less toxic than 10-MDP: in pulp stem cells, 10-MDP caused comparably greater viability loss [69]. Moreover, sub-cytotoxic levels of 10-MDP can also trigger pro-inflammatory cytokines and suppress odontoblastic differentiation [70], which may explain the lower viability observed with the G2 primer if any 10-MDP leaches out. By contrast, 4-META demonstrated high biocompatibility with rat pulp cells [71]. Our findings parallel a recent report in which G2 Bond Universal exhibited greater cytotoxic effects on pulp cells than a GPDM-based one-bottle adhesive—differences that the authors attributed to variations in adhesive chemistry [72]. Importantly, 15 vol% PA–ACP/MSN did not reduce viability: across 24–72 h, the filled and unfilled groups were statistically similar, indicating that the filler was biologically inert in this context. The nanofiller consists of mesoporous silica (≈50 nm) carrying polyacrylic-acid-stabilized amorphous calcium phosphate, and each component is expected to be benign. MSNs are widely regarded as safe carriers with tunable porosity and high cytocompatibility [73,74,75]. Consistently, Luo et al. reported that a similar PA–ACP-loaded expanded pore MSNs system was highly biocompatible, causing no significant cytotoxicity to dental pulp cells even at high concentrations [30].

Taken together, the results demonstrate that 15 vol% PA–ACP/MSN maintains fibroblast viability while preserving the intended remineralization potential. All groups exceeded the ISO threshold, indicating that the nanofiller can be integrated without the loss of biocompatibility. This validation supports clinical translation while meeting biocompatibility standards; nonetheless, interfacial remineralization must be demonstrated before any claims of remineralization benefits can be made.

## 5. Conclusions

In summary, we effectively synthesized amine-functionalized mesoporous silica nanoparticles loaded with PA-stabilized ACP and incorporated this hybrid nanofiller into two-step universal dental adhesives. The modified primers with up to 15 vol% PA–ACP/MSN retained acceptable wettability and produced resin–dentin bond strengths statistically equivalent to those of the unmodified controls. The mesoporous silica carrier preserved the high surface area and porosity necessary for significant PA–ACP loading, consistent with the potential capacity to release Ca^2+^ and PO_4_^3−^ ions without adversely affecting the adhesive’s bonding properties. All tested formulations demonstrated cell viability above 70%, indicating no cytotoxic concern due to the nanoparticle addition. However, at 20 vol% nanofiller, clear signs of overloading were observed—namely, increased contact angle and reduced bond strength—highlighting that an optimal filler content (10–15 vol%) should not be exceeded. This pilot adhesive design study represents a first step toward biomimetic universal adhesives; confirmation of interfacial remineralization and durability under clinically relevant aging is a key direction for future work.

## Figures and Tables

**Table 1:** Contact angle (θ) and immediate μTBS (MPa) of G2 Bond (G2) and OptiBond eXTRa (OE) primers containing 0–20 vol% PA–ACP/MSN. Values are mean ± SD; fracture modes (A/M/C) follow μTBS.

Group	θ (°) ± SD	MPa ± SD (A/M/C)
G2-C	20.6° ± 2.9°	46.2 ± 6.9 (8/4/3)
G2-10%	23.4° ± 2.6°	43.2 ± 5.7 (7/2/6)
G2-15%	27° ± 2°	40.8 ± 5.8 (10/4/1)
G2-20%	41.8° ± 3.4° *	37.9 ± 6.2 (13/2/0) *
OE-C	9.6° ± 1.9°	44.6 ± 5.7 (9/5/1)
OE-10%	12.7° ± 1.1°	41.2 ± 8.6 (6/6/3)
OE-15%	15.4° ± 3.3°	39.1 ± 5.6 (11/4/0)
OE-20%	24.3° ± 2.5° *	33.6 ± 4.4 (10/4/1) *

* indicates *p* < 0.05 versus the respective control (one-way ANOVA, Dunnett). A = Adhesive failure; M = Mixed failure; and C = Cohesive failure.

## Data Availability

The data presented in this study are available on request from the corresponding author due to institutional embargo.

## Supplementary Materials

The following supporting information can be downloaded at https://www.mdpi.com/article/10.3390/polym17182501/s1: Table S1. Mean (± SD) cell-viability (%) of human periodontal-ligament fibroblasts (HPLF) after 24, 48, and 72 h exposure to adhesive extracts. Each extract was prepared by pooling six discs per group and tested in triplicate wells. Table S2. Two-way ANOVA examining the effects of adhesive type, and loading on cell viability percentage (%) of HPLF cell to adhesive discs elutes after 24 h immersion time. Table S3. Two-way ANOVA examining the effects of adhesive type, and loading on cell viability percentage (%) of HPLF cell to adhesive discs elutes after 48 h immersion time. Table S4. Two-way ANOVA examining the effects of adhesive type, and loading on cell viability percentage (%) of HPLF cell to adhesive discs elutes after 72 h immersion time.

## Abbreviations

The following abbreviations are used in this manuscript:

G2	G2 Bond Universal
OE	OptiBond eXTRa Universal
PA–ACP	Polyacrylic-acid-stabilized amorphous calcium phosphate
MSNs	Mesoporous silica nanoparticles
APTES	3-Aminopropyltriethoxysilane
HPLFs	Human periodontal ligament fibroblasts
μTBS	Microtensile bond strength
BJH	Barrett–Joyner–Halenda
HRTEM	High-resolution transmission electron microscopy
FFT	Fast Fourier transform
FTIR	Fourier-transform infrared spectroscopy
BET	Brunauer–Emmett–Teller
10-MDP	10-methacryloyloxydecyl dihydrogen phosphate
GPDM	Glycerol phosphate dimethacrylate
4-META	4-methacryloxyethy trimellitic anhydride

## References

[1] Tjäderhane L., Nascimento F.D., Breschi L., Mazzoni A., Tersariol I.L., Geraldeli S., Tezvergil-Mutluay A., Carrilho M.R., Carvalho R.M., Tay F.R. (2013). Optimizing dentin bond durability: Control of collagen degradation by matrix metalloproteinases and cysteine cathepsins. Dent. Mater..

[2] Mokeem L.S., Garcia I.M., Melo M.A. (2023). Degradation and failure phenomena at the dentin bonding interface. Biomedicines.

[3] Al-Qrimli A.F., Al-Shammam A.M.W. (2016). Comparative Evaluation of the Effect of Different Universal Adhesives and Bonding Techniques on the Marginal Gap of Class I Composite Restoration (a Sem Study). J. Baghdad Coll. Dent..

[4] Spencer P., Ye Q., Park J., Topp E.M., Misra A., Marangos O., Wang Y., Bohaty B.S., Singh V., Sene F. (2010). Adhesive/dentin interface: The weak link in the composite restoration. Ann. Biomed. Eng..

[5] Amin F., Fareed M.A., Zafar M.S., Khurshid Z., Palma P.J., Kumar N. (2022). Degradation and stabilization of resin-dentine interfaces in polymeric dental adhesives: An updated review. Coatings.

[6] Spagnuolo G. (2022). Bioactive dental materials: The current status. Materials.

[7] Mohammed D.R., Ibrahim A.I., Deb S. (2025). Combating white spot lesions via incorporation of remineralizing/antibacterial additives into orthodontic adhesives: A review. J. Baghdad Coll. Dent..

[8] Xu H.H., Moreau J.L., Sun L., Chow L.C. (2011). Nanocomposite containing amorphous calcium phosphate nanoparticles for caries inhibition. Dent. Mater..

[9] Ali N.A.M., Nissan L.M., Khamis A.H., Al-Taai N. (2024). Enamel demineralization around orthodontic brackets bonded with new bioactive composite (in-vitro study). J. Baghdad Coll. Dent..

[10] Sauro S., Osorio R., Watson T.F., Toledano M. (2012). Therapeutic effects of novel resin bonding systems containing bioactive glasses on mineral-depleted areas within the bonded-dentine interface. J. Mater. Sci. Mater. Med..

[11] Wang Z., Ouyang Y., Wu Z., Zhang L., Shao C., Fan J., Zhang L., Shi Y., Zhou Z., Pan H. (2018). A novel fluorescent adhesive-assisted biomimetic mineralization. Nanoscale.

[12] Cölfen H., Mann S. (2003). Higher-order organization by mesoscale self-assembly and transformation of hybrid nanostructures. Angew. Chem. Int. Ed..

[13] Gower L.B. (2008). Biomimetic model systems for investigating the amorphous precursor pathway and its role in biomineralization. Chem. Rev..

[14] Dey A., Bomans P.H., Müller F.A., Will J., Frederik P.M., de With G., Sommerdijk N.A. (2010). The role of prenucleation clusters in surface-induced calcium phosphate crystallization. Nat. Mater..

[15] Hua F., Yan J., Zhao S., Yang H., He H. (2020). In vitro remineralization of enamel white spot lesions with a carrier-based amorphous calcium phosphate delivery system. Clin. Oral Investig..

[16] Garma N.M., Ibrahim A.I. (2023). Development of a remineralizing calcium phosphate nanoparticle-containing self-etching system for orthodontic bonding. Clin. Oral Investig..

[17] Li Z., Zhang Y., Feng N. (2019). Mesoporous silica nanoparticles: Synthesis, classification, drug loading, pharmacokinetics, biocompatibility, and application in drug delivery. Expert Opin. Drug Deliv..

[18] Florek J., Caillard R., Kleitz F. (2017). Evaluation of mesoporous silica nanoparticles for oral drug delivery–current status and perspective of MSNs drug carriers. Nanoscale.

[19] Hussein E.A., Kareem S.H. (2021). Mesoporous silica nanoparticles as a system for ciprofloxacin drug delivery; kinetic of adsorption and releasing. Baghdad Sci. J..

[20] Choi Y., Sun W., Kim Y., Kim I.-R., Gong M.-K., Yoon S.-Y., Bae M.-K., Park B.-S., Park S.-B., Kim Y.-I. (2020). Effects of Zn-doped mesoporous bioactive glass nanoparticles in etch-and-rinse adhesive on the microtensile bond strength. Nanomaterials.

[21] Choi A., Yoo K.-H., Yoon S.-Y., Park B.-S., Kim I.-R., Kim Y.-I. (2021). Anti-microbial and remineralizing properties of self-adhesive orthodontic resin containing mesoporous bioactive glass. Materials.

[22] Alkhazaleh A., Elfagih S., Chakka L.R.J., Armstrong S.R., Comnick C.L., Qian F., Salem A.K., Guymon C.A., Haes A.J., Vidal C.M. (2022). Development of proanthocyanidin-loaded mesoporous silica nanoparticles for improving dental adhesion. Mol. Pharm..

[23] Möller K., Kobler J., Bein T. (2007). Colloidal Suspensions of Nanometer-Sized Mesoporous Silica. Adv. Funct. Mater..

[24] Kobler J., Möller K., Bein T. (2008). Colloidal suspensions of functionalized mesoporous silica nanoparticles. ACS Nano.

[25] Kresge a.C., Leonowicz M.E., Roth W.J., Vartuli J., Beck J. (1992). Ordered mesoporous molecular sieves synthesized by a liquid-crystal template mechanism. Nature.

[26] Zhang W., Luo X.-J., Niu L.-N., Yang H.-Y., Yiu C.K., Wang T.-D., Zhou L.-Q., Mao J., Huang C., Pashley D.H. (2015). Biomimetic intrafibrillar mineralization of type I collagen with intermediate precursors-loaded mesoporous carriers. Sci. Rep..

[27] Slowing I.I., Vivero-Escoto J.L., Wu C.-W., Lin V.S.-Y. (2008). Mesoporous silica nanoparticles as controlled release drug delivery and gene transfection carriers. Adv. Drug Deliv. Rev..

[28] Maleki A., Kettiger H., Schoubben A., Rosenholm J.M., Ambrogi V., Hamidi M. (2017). Mesoporous silica materials: From physico-chemical properties to enhanced dissolution of poorly water-soluble drugs. J. Control. Release.

[29] Yang H.-Y., Niu L.-N., Sun J.-L., Huang X.-Q., Pei D.-D., Huang C., Tay F.R. (2017). Biodegradable mesoporous delivery system for biomineralization precursors. Int. J. Nanomed..

[30] Luo X.-J., Yang H.-Y., Niu L.-N., Mao J., Huang C., Pashley D.H., Tay F.R. (2016). Translation of a solution-based biomineralization concept into a carrier-based delivery system via the use of expanded-pore mesoporous silica. Acta Biomater..

[31] Gao L., Sun J., Li Y., Zhang L. (2011). Bimodal mesoporous silicas functionalized with different level and species of the amino groups for adsorption and controlled release of aspirin. J. Nanosci. Nanotechnol..

[32] Zong C., Wang Y., Jiang S. (2023). Toward the understanding of poly (Acrylic Acid) on amorphous calcium phosphate mediated collagen intrafibrillar Mineralization: Surface adsorption versus bulk incorporation. J. Cryst. Growth.

[33] Wang J., Chen Y., Li L., Sun J., Gu X., Xu X., Pan H., Tang R. (2013). Remineralization of dentin collagen by meta-stabilized amorphous calcium phosphate. CrystEngComm.

[34] Qi Y., Ye Z., Fok A., Holmes B.N., Espanol M., Ginebra M.-P., Aparicio C. (2018). Effects of molecular weight and concentration of poly (acrylic acid) on biomimetic mineralization of collagen. ACS Biomater. Sci. Eng..

[35] Zhao X.S., Lu G., Whittaker A., Millar G., Zhu H. (1997). Comprehensive study of surface chemistry of MCM-41 using 29Si CP/MAS NMR, FTIR, pyridine-TPD, and TGA. J. Phys. Chem. B.

[36] Zhao D., Huo Q., Feng J., Chmelka B.F., Stucky G.D. (1998). Nonionic triblock and star diblock copolymer and oligomeric surfactant syntheses of highly ordered, hydrothermally stable, mesoporous silica structures. J. Am. Chem. Soc..

[37] Vallet-Regi M., Rámila A., Del Real R., Pérez-Pariente J. (2001). A new property of MCM-41: Drug delivery system. Chem. Mater..

[38] Wei S., Wu H., Luo X.-J. (2020). Biomineralization precursor carrier system based on carboxyl-functionalized large pore mesoporous silica nanoparticles. Curr. Med. Sci..

[39] Chen C., Weir M.D., Cheng L., Lin N.J., Lin-Gibson S., Chow L.C., Zhou X., Xu H.H. (2014). Antibacterial activity and ion release of bonding agent containing amorphous calcium phosphate nanoparticles. Dent. Mater..

[40] Melo M.A.S., Cheng L., Zhang K., Weir M.D., Rodrigues L.K., Xu H.H. (2013). Novel dental adhesives containing nanoparticles of silver and amorphous calcium phosphate. Dent. Mater..

[41] Adabo G.L., dos Santos Cruz C.A., Fonseca R.G., Vaz L.s.G. (2003). The volumetric fraction of inorganic particles and the flexural strength of composites for posterior teeth. J. Dent..

[42] Balkaya H., Demirbuğa S. (2023). Evaluation of six different one-step universal adhesive systems in terms of dentin bond strength, adhesive interface characterization, surface tension, contact angle, degree of conversion and solvent evaporation after immediate and delayed use. J. Esthet. Restor. Dent..

[43] Genari B., Leitune V.C.B., Jornada D.S., Aldrigui B.R., Pohlmann A.R., Guterres S.S., Samuel S.M.W., Collares F.M. (2018). Effect on adhesion of a nanocapsules-loaded adhesive system. Braz. Oral Res..

[44] Armstrong S., Breschi L., Özcan M., Pfefferkorn F., Ferrari M., Van Meerbeek B. (2017). Academy of Dental Materials guidance on in vitro testing of dental composite bonding effectiveness to dentin/enamel using micro-tensile bond strength (μTBS) approach. Dent. Mater..

[45] (2023). Dentistry—Test Methods for Tensile Bond Strength to Tooth Structure.

[46] (2009). Biological Evaluation of Medical Devices—Part 5: Tests For In Vitro Cytotoxicity.

[47] (2025). Dentistry—Evaluation of Biocompatibility of Medical Devices Used in Dentistry.

[48] Alhashimi R.A., Mannocci F., Sauro S. (2017). Bioactivity, cytocompatibility and thermal properties of experimental Bioglass-reinforced composites as potential root-canal filling materials. J. Mech. Behav. Biomed. Mater..

[49] Catunda R.-Q., de Oliveira E.-B., da Silva E.-C., Brasil V.-L.-M. (2017). Citotoxicity evaluation of three dental adhesives on vero cells in vitro. J. Clin. Exp. Dent..

[50] Franz A., König F., Lucas T., Watts D.C., Schedle A. (2009). Cytotoxic effects of dental bonding substances as a function of degree of conversion. Dent. Mater..

[51] Sun F., Liu Y., Pan Y., Chen M., Meng X. (2018). Cytotoxicity of Self-Adhesive Resin Cements on Human Periodontal Ligament Fibroblasts. BioMed Res. Int..

[52] Al-Labban Y.R., Alrubayee M., Zaidi S.J.A., Kazmi S. (2024). Effects of Adding Tricalcium Silicate Nanoparticles to the Universal G2 Bond Adhesive as Self-Etch Mode on the Shear Bond Strength to the Orthodontic Bracket. Clin. Exp. Dent. Res..

[53] Thommes M., Kaneko K., Neimark A.V., Olivier J.P., Rodriguez-Reinoso F., Rouquerol J., Sing K.S. (2015). Physisorption of gases, with special reference to the evaluation of surface area and pore size distribution (IUPAC Technical Report). Pure Appl. Chem..

[54] Gregg S.J., Sing K.S.W., Salzberg H. (1967). Adsorption surface area and porosity. J. Electrochem. Soc..

[55] Zhao D., Feng J., Huo Q., Melosh N., Fredrickson G.H., Chmelka B.F., Stucky G.D. (1998). Triblock copolymer syntheses of mesoporous silica with periodic 50 to 300 angstrom pores. Science.

[56] Trewyn B.G., Slowing I.I., Giri S., Chen H.-T., Lin V.S.-Y. (2007). Synthesis and functionalization of a mesoporous silica nanoparticle based on the sol–gel process and applications in controlled release. Acc. Chem. Res..

[57] Barrett E.P., Joyner L.G., Halenda P.P. (1951). The determination of pore volume and area distributions in porous substances. I. Computations from nitrogen isotherms. J. Am. Chem. Soc..

[58] Xiong L., Du X., Shi B., Bi J., Kleitz F., Qiao S.Z. (2015). Tunable stellate mesoporous silica nanoparticles for intracellular drug delivery. J. Mater. Chem. B.

[59] He Y., Luo L., Liang S., Long M., Xu H. (2017). Amino-functionalized mesoporous silica nanoparticles as efficient carriers for anticancer drug delivery. J. Biomater. Appl..

[60] Niu L.-N., Zhang W., Pashley D.H., Breschi L., Mao J., Chen J.-H., Tay F.R. (2014). Biomimetic remineralization of dentin. Dent. Mater..

[61] Pires-de F.d.C.P., Tonani-Torrieri R., Vivanco R.G., de Arruda C.N.F., Geraldeli S., Sinhoreti M.A.C., Roulet J.-F. (2022). Effect of incorporation of bioactive glass-ceramic into self-etch adhesives. J. Adhes. Dent..

[62] Han R., Zhang Z., Zhao Y., Yan L., Tang W., Zhang H. (2025). Preparation and performance evaluation of Zn-N-TiO2 nanoparticles-containing dental adhesive. Polym. Test..

[63] Yan H., Wang S., Han L., Peng W., Yi L., Guo R., Liu S., Yang H., Huang C. (2018). Chlorhexidine-encapsulated mesoporous silica-modified dentin adhesive. J. Dent..

[64] Melo M.A.S., Cheng L., Weir M.D., Hsia R.C., Rodrigues L.K., Xu H.H. (2013). Novel dental adhesive containing antibacterial agents and calcium phosphate nanoparticles. J. Biomed. Mater. Res. Part B Appl. Biomater..

[65] Yang Y., Ding Y., Fan Y., Ren L., Tang X., Meng X. (2021). Application of silver nanoparticles in situ synthesized in dental adhesive resin. Int. J. Adhes. Adhes..

[66] Demirci M., Hiller K.-A., Bosl C., Galler K., Schmalz G., Schweikl H. (2008). The induction of oxidative stress, cytotoxicity, and genotoxicity by dental adhesives. Dent. Mater..

[67] Caldas I.P., Alves G.G., Barbosa I.B., Scelza P., de Noronha F., Scelza M.Z. (2019). In vitro cytotoxicity of dental adhesives: A systematic review. Dent. Mater..

[68] Demirel G., Demirsoy F.F.K., Irmak Ö. (2020). Cytotoxicity evaluation of eluates from universal adhesives by real-time cell analysis. Dent. Mater. J..

[69] Cavallaro-Mota F.D., Esposo G.N., Kury M., Fronza B.M., Saraceni C.H.C., Andia D.C., Lima A.F. (2025). Assessment of 10-MDP and GPDM monomers on viability and inflammatory response in human dental pulp stem cells. Dent. Mater..

[70] Kim E.C., Park H., Lee S.I., Kim S.Y. (2015). Effect of the acidic dental resin monomer 10-methacryloyloxydecyl dihydrogen phosphate on odontoblastic differentiation of human dental pulp cells. Basic Clin. Pharmacol. Toxicol..

[71] Nakagawa K., Saita M., Ikeda T., Hirota M., Park W., Lee M.C.-I., Ogawa T. (2015). Biocompatibility of 4-META/MMA-TBB resin used as a dental luting agent. J. Prosthet. Dent..

[72] Ersöz B., Aydin N., Oktay E.A., Çal İ.K., Karaoğlanoğlu S. (2025). Effects of universal adhesives on dentin matrix proteins, matrix metalloproteinases and cytokine release of human pulp cells. Odontology.

[73] Asefa T., Tao Z. (2012). Biocompatibility of mesoporous silica nanoparticles. Chem. Res. Toxicol..

[74] Tarn D., Ashley C.E., Xue M., Carnes E.C., Zink J.I., Brinker C.J. (2013). Mesoporous silica nanoparticle nanocarriers: Biofunctionality and biocompatibility. Acc. Chem. Res..

[75] Tang F., Li L., Chen D. (2012). Mesoporous silica nanoparticles: Synthesis, biocompatibility and drug delivery. Adv. Mater..

