# Supplementary material for: Biomimetic Filler Strategy for Two-Step Universal Dental Adhesives Using PA–ACP/MSN: Effects on Wettability, Immediate Microtensile Bond Strength, and Cytocompatibility
Source: Polymers (Basel). 2025 Sep 16;17(18):2501. doi: 10.3390/polym17182501 (PMC12473806; doi:10.3390/polym17182501)
Supplement: Supplementary file 1 [file polymers-17-02501-s001.zip › polymers-3851296-supplementary.pdf]

Table S1. Mean (± SD) cell-viability (%) of human periodontal-ligament fibroblasts (HPLF) after 24, 48, and 72 h exposure to adhesive extracts. Each extract was prepared by pooling six discs per group and tested in triplicate wells.

Immersion time	Group	Mean Viability (SD)
24 h	OE-C	92.419 (1.754)
	OE-15%	90.683 (1.647)
	G2-C	88.522 (1.886)
	G2-15%	87.423 (1.746)
48 h	OE-C	87.153 (2.140)
	OE-15%	86.208 (1.894)
	G2-C	84.161 (1.945)
	G2-15%	82.870 (2.228)
72 h	OE-C	84.606 (1.881)
	OE-15%	83.430 (1.688)
	G2-C	81.829 (2.146)
	G2-15%	80.421 (2.142)

Table S2. Two-way ANOVA examining the effects of Adhesive Type, and Loading on Cell viability percentage (%) of HPLF cell to adhesive discs elutes after 24 h immersion time.

Source	Type III Sum of Squares	df	Mean Square	F	Sig.
Corrected Model	89.495	3	29.832	8.023	.001
Intercept	193372.195	1	193372.195	52007.651	.000
Adhesive type	76.826	1	76.826	20.662	.000
Loading	12.061	1	12.061	3.244	.087
Adhesive Type * Nano-filler Loading	.608	1	.608	.163	.690
Error	74.363	20	3.718		
Total	193536.054	24			
Corrected Total	163.858	23			

Table S3. Two-way ANOVA examining the effects of Adhesive Type, and Loading on Cell viability percentage (%) of HPLF cell to adhesive discs elutes after 48 h immersion time.

Source	Type III Sum of Squares	df	Mean Square	F	Sig.
Corrected Model	67.763	3	22.588	4.451	.015
Intercept	173799.651	1	173799.651	34244.547	.000
Adhesive type	60.086	1	60.086	11.839	.003
Loading	7.498	1	7.498	1.477	.238
Adhesive Type * Nano-filler loading	.179	1	.179	.035	.853
Error	101.505	20	5.075		
Total	173968.919	24			
Corrected Total	169.268	23			

Table S4. Two-way ANOVA examining the effects of Adhesive Type, and Loading on Cell viability percentage (%) of HPLF cell to adhesive discs elutes after 72 h immersion time.

Source	Type III Sum of Squares	df	Mean Square	F	Sig.
Corrected Model	60.337	3	20.112	4.303	.017
Intercept	163632.761	1	163632.761	35012.048	.000
Adhesive type	50.235	1	50.235	10.749	.004
Loading	10.022	1	10.022	2.144	.159
Adhesive Type * Nano-filler loading	.080	1	.080	.017	.897
Error	93.472	20	4.674		
Total	163786.571	24			
Corrected Total	153.810	23			
